# Sexual behaviour, recreational drug use and hepatitis C co-infection in HIV-diagnosed men who have sex with men in the United Kingdom: results from the ASTRA study

**DOI:** 10.7448/IAS.17.4.19630

**Published:** 2014-11-02

**Authors:** Marina Daskalopoulou, Alison Rodger, Alicia Thornton, Andrew Phillips, Lorraine Sherr, Richard Gilson, Margaret Johnson, Martin Fisher, Jane Anderson, Jeffrey McDonnell, Simon Edwards, Nicky Perry, Simon Collins, Sanjay Bhagani, Andrew Speakman, Colette Smith, Fiona Lampe

**Affiliations:** 1 University College London Royal Free Hospital, London, UK; 2 Royal Free Hospital, London, UK; 3 Brighton and Sussex University Hospitals, Brighton, UK; 4 Homerton University Hospitals, London, UK; 5 Central and North West London, London, UK; 6 HIVi-Base, London, UK

## Abstract

**Introduction:**

Transmission of Hepatitis C virus (HCV) among HIV-positive men who have sex with men (MSM) in the United Kingdom is ongoing. We explore associations between self-reported sexual behaviours and drug use with cumulative HCV prevalence, as well as new HCV diagnosis.

**Methods:**

ASTRA is a cross-sectional questionnaire study including 2,248 HIV-diagnosed MSM under care in the United Kingdom during 2011–2012. Socio-demographic, lifestyle, HIV-related and sexual behaviour data were collected during the study. One thousand seven hundred and fifty two (≥70%) of the MSM who consented to linkage of ASTRA and clinical information (prior to and post questionnaire) were included. Cumulative prevalence of HCV was defined as any positive anti-HCV or HCV-RNA test result at any point prior to questionnaire completion. We excluded 536 participants with clinical records only after questionnaire completion. Among the remaining 1,216 MSM, we describe associations of self-reported sexual behaviours and recreational drug use in the three months prior to ASTRA with cumulative HCV prevalence, using modified Poisson regression with robust error variances. New HCV was defined as any positive anti-HCV or HCV-RNA after questionnaire completion. We excluded 591 MSM who reported ever having a HCV diagnosis at questionnaire, any positive HCV result prior to questionnaire or did not have any HCV tests after the questionnaire. Among the remaining 1,195 MSM, we describe occurrence of new HCV diagnosis during follow-up according to self-reported sexual behaviours and recreational drug use three months prior to questionnaire (Fisher's exact test).

**Results:**

Cumulative HCV prevalence among MSM prior to ASTRA was 13.3% (95% CI 11.5–15.4). Clinic- and age-adjusted prevalence ratios (95% CI) for cumulative HCV prevalence were 4.6 (3.1–6.7) for methamphetamine, 6.5 (3.5–12.1) for injection drugs, 2.3 (1.6–3.4) for gamma hydroxybutyrate (GHB), 1.6 (1.3–2.0) for nitrites, 1.7 (1.5–2.0) for all condom-less sex (CLS), 2.1 (1.7–2.5) for CLS-HIV-seroconcordant, 1.3 (0.9–1.9) for CLS-HIV-serodiscordant, 2.0 (1.6–2.5) for group sex, 1.5 (1.2–1.9) for more than 10 new sexual partners in the past year. Among 1,195 MSM with 2.2 years [IQR 1.5–2.4] median follow-up, there were 7 new HCV cases during 2,033 person-years at risk. Incidence was 3.5 per 1,000 person-years (95% CI 1.6–7.2). New HCV was recorded in 1.3% MSM who used methamphetamine versus 0.5% MSM who did not (p=0.385); 3.7% MSM who injected recreational drugs versus 0.5% MSM who did not (p=0.148); 2.9% MSM who used GHB versus 0.4% MSM who did not (p=0.003); 1.5% MSM who used nitrites versus 0.2% MSM who did not (p=0.019); 1.1% MSM having CLS versus 0.3% MSM who did not (p=0.084); 1.7% MSM having CLS-HIV-serodiscordant versus 0.4% MSM who did not (p=0.069); 0.9% MSM who had CLS-HIV-seroconcordant versus 0.5% MSM who did not (p=0.318); 0.8% MSM who had group sex versus 0.5% MSM who did not (p=0.463); and 1.6% MSM with =10 new sexual partners in the previous year versus 0.2% MSM with no or up to 9 new partners (p=0.015).

**Conclusions:**

Self-reported recent use of recreational and injection drugs, condom-less sex and multiple new sexual partners are associated with pre-existing HCV infection and, with the exception of injection drugs, appear to be predictive of new HCV co-infection among HIV-diagnosed MSM.

